# *Clostridium* species as probiotics: potentials and challenges

**DOI:** 10.1186/s40104-019-0402-1

**Published:** 2020-02-20

**Authors:** Pingting Guo, Ke Zhang, Xi Ma, Pingli He

**Affiliations:** State Key Laboratory of Animal Nutrition, China Agricultural University, No. 2 Yuanmingyuan West Road, Beijing, 100193 China

**Keywords:** *Clostridium* species, Intestinal homeostasis, Metabolites, Probiotics

## Abstract

*Clostridium* species, as a predominant cluster of commensal bacteria in our gut, exert lots of salutary effects on our intestinal homeostasis. Up to now, *Clostridium* species have been reported to attenuate inflammation and allergic diseases effectively owing to their distinctive biological activities. Their cellular components and metabolites, like butyrate, secondary bile acids and indolepropionic acid, play a probiotic role primarily through energizing intestinal epithelial cells, strengthening intestinal barrier and interacting with immune system. In turn, our diets and physical state of body can shape unique pattern of *Clostridium* species in gut. In view of their salutary performances, *Clostridium* species have a huge potential as probiotics. However, there are still some nonnegligible risks and challenges in approaching application of them. Given this, this review summarized the researches involved in benefits and potential risks of *Clostridium* species to our health, in order to develop *Clostridium* species as novel probiotics for human health and animal production.

## Background

The gastrointestinal tract inhabits lots of bacteria [1–4]. Species of *Clostridium* cluster XIVa and IV, as the representatives of the predominant bacteria in gut, account for 10–40% of the total bacteria [5]. They are well-known as the indispensable regulators of intestinal homeostasis. It was reported that species of *Clostridium* clusters XIVa and IV were essential for normalization of germfree mice [6]. In ulcerative colitis, *Clostridium butyricum* (*C*. *butyricum*) and *Eubacterium rectale* were associated with low clinical activity indices [7]. The count of *Clostridium* clusters III, IV, and XIVa species also reduced in intestinal failure [8]. Furthermore, *Clostridium* species are potent candidates to alleviate dysfunctions and disorders in intestine. The ameliorative effects of colitis and allergic diarrhea were observed through oral administration of 17 strains belonging to *Clostridium* clusters IV, XIVa and XVIII [9]. But it should be noted that there is still safety concern about the exotoxin secretion of some *Clostridium* species, like alpha-toxin and enterotoxin from *Clostridium perfringens* (*C. perfringens*), toxin A and toxin B from *Clostridium difficile* (*C. difficile*) [10, 11]. Meanwhile, the efficiency of *Clostridium* species must be considered when applied to animal production and diseases treatment. So this review summarized the reports about both the benefits and underlying risks from *Clostridium* species on intestinal immune regulation and disease prevention to elucidate the potentials and challenges of their novel roles as probiotic.

## The taxonomy of genus *Clostridium*

The bacteria of genus *Clostridium* are rod-shaped, gram-positive and spore-forming anaerobes. They distribute in soil, intestinal tract of animals, water and other biotopes. At the outset, the bacteria were classified into genus *Clostridium* based on the morphological and physiological characteristics above. But with the further in-depth studies of *Clostridium* species, the heterogeneities among them become more and more noteworthy. Twenty years ago, researchers put forward a novel taxonomic arrangement criterion on the strength of phylogenetic analyses of 16S rRNA gene sequences [6, 12]. The genus *Clostridium* was classified into 19 clusters. The new criterion introduced some asporulate bacteria, like *Roseburia cecicola* and *Ruminococcus torques*. And most previous members of *Clostridium* were assigned to *Clostridium* cluster I, represented by *C. butyricum*. The *Clostridium* species discussed in this review is based on this new criterion.

## Distribution and colonization of *Clostridium* species in gut

### Distribution

**Fig. 1:**
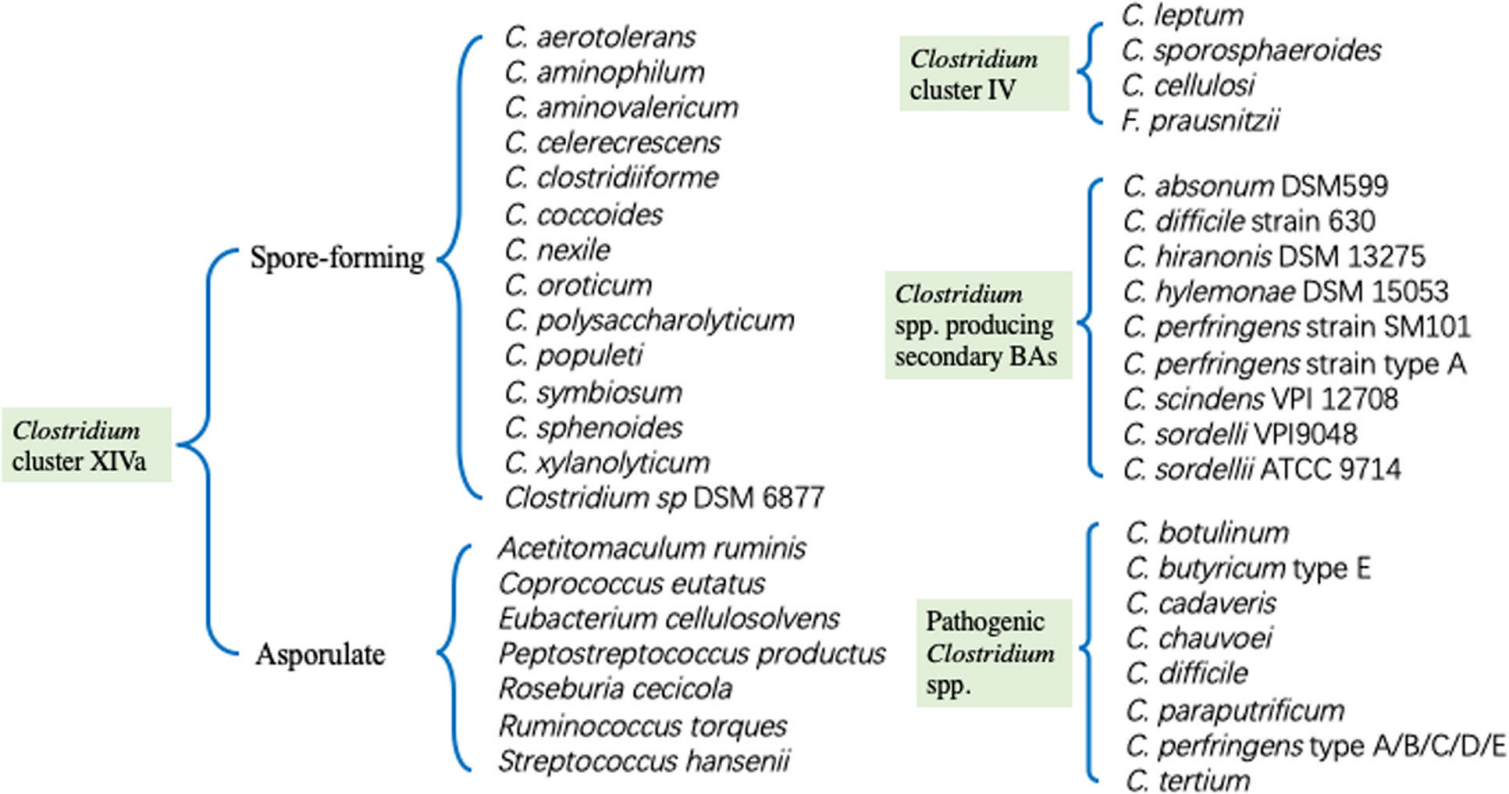
*Clostridium* cluster IV and XIVa species, *Clostridium* spp. producing secondary BAs and pathogenic *Clostridium* spp.. According to the novel taxonomic arrangement criterion, the species of *Clostridium* cluster IV and XIVa are listed. What’s more, *Clostridium* spp. that can convert primary BAs to secondary BAs are presented here. Pathogenic *Clostridium* spp. listed in Fig. 1 also have some non-pathogenic strains, and most of them are commensal bacteria in gut.

In the intestine of human and animals, *Clostridium* species, as one of the richest bacterial cluster, are mainly composed of *Clostridium* cluster IV and XIVa (Fig. 1). *Clostridium* cluster IV, also called *C. leptum* group, have 4 members, namely *C. leptum*, *C. sporosphaeroides*, *C. cellulosi* and *Faecalibacterium prausnitzii (F. prausnitzii). Clostridium* cluster XIVa, also known as *Clostridium coccoides* group, consists of 21 species. Except *Clostridium* spp., *Acetitomaculum ruminis*, *Roseburia cecicola*, *Coprococcus eutactus*, *Ruminococcus torques*, *Streptococcus hansenii* and *Eubacterium cellulosolvens* are also included in *Clostridium* species [5, 12]. *Clostridium* species can utilize large amounts of nutrients that cannot be digested by host and produce lots of short-chain fatty acids (SCFAs), which play a noticeable role in intestinal homeostasis. Generally, *Clostridium* species predominate in large intestine, especially in the mucosal folds of ascending colon, living in harmony with Bacteroidaceae, Enterococcaceae and Lactobacillaceae, which colonize in colonic lumen [5].

### Colonization

Clostridia are one of members of early-colonized bacteria and they could be detected in feces within the first week of birth. Most of them are *C. butyricum*, *C. paraputrificum* and *C. difficile*. It is interesting that these *Clostridium* species existed consistently from birth to 1 year old in the formula-fed infant, but dismissed in breast-fed infant after weaning [13]. And the *Clostridium* pattern in infants was also distinct from adults, with higher proportion of *Clostridium* cluster I in infants but higher *Clostridium* cluster IV and XIVa in adults. Similar to human, *Clostridium* species were also found in the feces of calves during the first postnatal week [14].

However, the phenomenons above do not imply that *Clostridium* species can stably inhabit in intestine. A research group investigated the intestinal colonization of *C. butyricum* strain CBM588. The spores of CBM588 were orally administrated into the Wistar rats. More than 10 times of viable spores were detected in small intestine 30 min after administration and vegetative cells of *C. butyricum* appeared in distal small intestine 2 h later. 5 h later, vegetative cells existed in cecum and colon. But *C. butyricum* disappeared in intestine 3 d after administration [15]. It means that *C. butyricum* strain CBM588 germinated and grew but didn’t colonize in intestine. However, the successful germination and growth of *C. butyricum* in intestine is in accordance with some *in vitro* experiments which showed that the spores of *C. butyricum* germinated and grew in the medium with Eh of +330 mV and a liquid paraffin covering [16]. These phenomenons can be explained by the active oxygen species scavenging ability of *C. butyricum*. *C. butyricum* was reported to grow at its anaerobic growth rate after consumption all of the dissolved oxygen in the medium, because *C. butyricum* possessed NADH/NADPH peroxidase and uperoxide dismutase, which were distributed widely in the genus *Clostridium* [17]. Different from *C. butyricum*, *F. prausnitzii* could take advantage of another mechanism to eliminate active oxygen species. *F. prausnitzii* possessed an extracellular electron shuttle, which contributes to *F. prausnitzii* growing at oxic-anoxic interphases, for example, the surface of colonic epithelial [18]. Except that, *F. prausnitzii* was reported to steadily prime in colon with the help of *Escherichia coli* colonization in small intestine [19]. As for *C. butyricum*, only some *in vitro* experiments suggested its adhesion to the surface of epithelial cells and its inhibition of pathogens adhesion, in spite of its strong adaptability to anaerobic environment [20].

As a whole, the ability of colonization in intestine vary a lot between *Clostridium* species and strains. Theoretically, bacterial adhesion will tremendously contribute to its colonization and predominance in colon. Hence, more high-adhesion *Clostridium* species are worthy of more in-depth researches to discover.

## Health benefits from *Clostridium* species

As the predominant bacteria in gut, *Clostridium* species exert lots of benefits to body health via interacting with intestine directly or indirectly. Thus, we will pay more attention on the benefits to gut health from *Clostridium* species in this section to clarify their concrete probitic effects. Herein, direct interaction with immune system and production of metabolites are two main pathways for *Clostridium* species to play a role in gut health.

### Benefits from crosstalk between *Clostridium* species and intestinal immune system

Most *Clostridium* species are the commensal bacteria and live in harmony with the intestinal environment. The underlying mechanism on immune tolerance of *Clostridium* species are being uncovered gradually with more and more in-depth studies. Hereinto, *F. prausnitzii* is a high-profile representative of *Clostridium* species in recent studies.

**Fig. 2:**
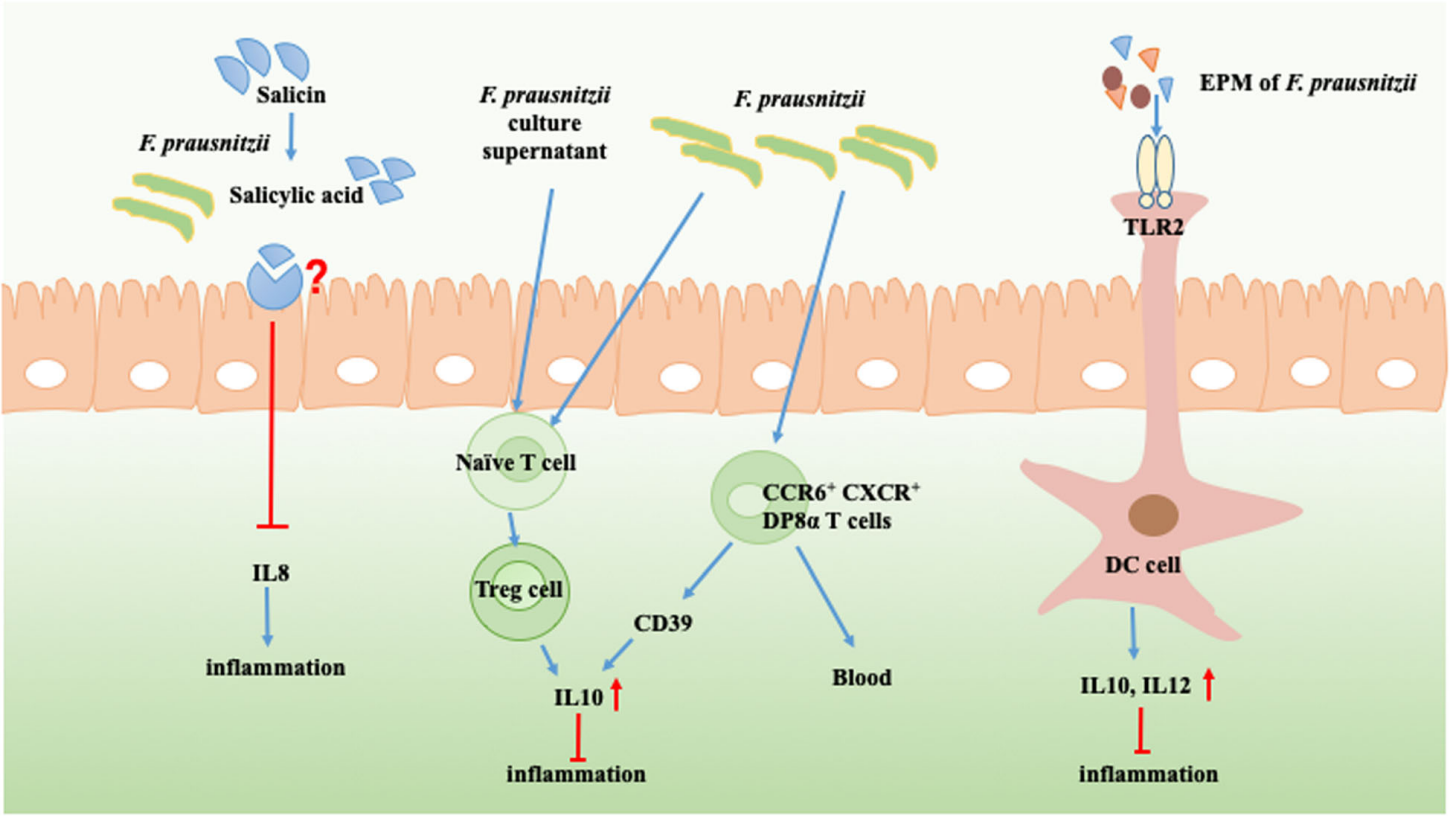
The interaction between *F. prausnitzii* and colonic immune. *F. prausnitzii* could exert anti-inflammation effects to our health dependent on its interaction with colonic immune to a great extent. 1) Salicylic acid could be produced from salicin fermentation by 40% *F. prausnitzii* and block the production of IL8 [28]. 2) *F. prausnitzii* and its culture supernatant could exhibit anti-inflammatory effects via IL10 production from Treg cells [19]. 3) CCR6^+^ CXCR6^+^ DP8α T cells are a new gut-derived T_REG_ cell subpopulation. They can particularly response to *F. prausnitzii* and exert anti-inflammation effect by promoting the IL10 production dependent on CD39 [23]. 4) Extracellular polymeric matrix (EPM) of *F. prausnitzii* strain HTF-F induced the TLR2-dependent secretion of IL10 and IL12 in human monocyte-derived dendritic cells (DC cells) to attenuate inflammation [24]

In a study conducted in 2008, *F. prausnitzii* was reported to protect from inflammation *in vitro* and *in vivo* through blocki*ng* NF*-κ*B activation and IL8 production [21]. Meanwhile, both *F. prausnitzii* and its culture supernatant could exhibit anti-inflammatory effects under recovery from chronic colitis and colitis reactivation [22–24]. Umesaki and his colleagues found that a defined mixture of 46 strains of *Clostridium* species belonging to *Clostridium* clusters XIVa and IV could modify the intraepithelial lymphocytes profile in large intestine [19]. Another research proposed that clusters IV and XIVa of the genus *Clostridium* promoted mucosal Treg cell accumulation in colon and a cocktail of 46 *Clostridium* strains could enrich transforming growth factor-β in colon [25]. Similarly, the 17 strains belonging to clusters IV, XIVa and XVIII of *Clostridia* induced the expansion and differentiation of Treg cells and oral administration of them could attenuate colitis and allergic diarrhea of mice [9]. A recent study discovered a new gut-derived T_REG_ cell subpopulation, named DP8α, which could express both CD4 and CD8α. Among DP8α T cells, there were *F.* prau-specific T cells co-expressing CCR6 and CXCR6, decreased in inflammatory bowel disease (IBD) patients [26]. But the results haven’t been verified in animals. These researches above suggested that *Clostridium* species could powerfully improve gut immune tolerance (Fig. 2).

To further explore the mechanisms of *Clostridium*-immune interaction, a research group isolated the extracellular polymeric matrix (EPM) of *F. prausnitzii* strain HTF-F and found it could form biofilm. At the same time, EPM could induce the TLR2-dependent secretion of IL10 and IL12 to attenuate inflammation [27]. It was proposed that *Escherichia coli* colonization in small intestine facilitated the colonization of *F. prausnitzii* in colon [19]. So gnotobiotic mice harboring *F. prausnitzii* and *Escherichia coli* were utilized as model to reveal the anti-inflammation mechanisms of *F. prausnitziiin vivo*. The results showed that salicylic acid directly assisted *F. prausnitzii* to withstand inflammation. Salicylic acid could be produced from salicin fermentation by 40% *F. prausnitzii* and block the production of IL8 [28] (Fig. 2). Hence, the benefit to health from *F. prausnizii *may attribute to their components and metabolites.

### Metabolites of *Clostridium* species and their benefits for gut health

*Clostridium* species are chemoorganotrophic bacteria. They can ferment a variety of nutrients, like carbohydrate, protein, organic acid and other organics, to produce acetic acid, propionic acid, butyric acid, and some solvents, such as acetone and butanol. In intestine of animals and human, *Clostridium* species mostly utilize indigestible polysaccharide. And most of the metabolites they produced bring out many benefits to gut health.

#### SCFAs

*Clostridium* species, along with some species belonging to Ruminaceae and Lachnospiraceae, are the main forces to generate short-chain fatty acids (SCFAs) from carbohydrate fermentation. SCFAs, particularly butyrate, as outstanding conductors, orchestrate multiple physiological functions to optimize luminal environment and maintain intestinal health.

Acetate can be the co-substrate used by cross-feeding bacteria to produce butyrate and possesses partial physiological functions of butyrate [29]. Propionate is utilized mostly by liver and involved in regulation of glucose and lipid metabolism [29]. Among SCFAs, butyrate is the most multifunctional and we will discuss its production in intestine and impacts on gut health in detail below.

There are 2 main metabolic pathways for bacteria in intestine to release butyrate. One is the butyryl-CoA transferase pathway, which is predominant and formed by various bacteria of *Clostridium*, such as *F. prausnitzii*, *Coprococcus eutactus* and *Roseburia* species. Another is the butyrae kinase pathway, which is dominative in *C. butyricum*, *Coproccus eutactus*, *Coprococcus comes* and so on. Four key enzymes are critical in conventing acety-CoA to butyrate, namely thiolase, 3-hydroxybutyrylCoA dehydrogenase, phosphotransbutyrylase and butyrate kinase [30]. Additionally, the catabolism of some amino acids (AAs) like lysine also produce butyrate [30, 31]. So the expression levels of *but* and *buk* genes (coding butyryl-CoA transferase and butyrate kinase respectively) have strong positive correction with the content of luminal butyrate and the amount of butyrate-producing bacteria in gut.

Nowadays, overwhelming evidence suggests the benefits from butyrate to intestinal health [32, 33]. Butyrate acts as the preferred energy source for colonic epithelial cells, exerts anti-inflammation effects, decreases the luminal pH to reduce bile salt solubility, inhibits ammonia absorption, hampers the invasion of pathogens and so forth. These aforementioned functions of butyrate have been illuminated in detail in a review published in 2016 [29]. And more novel progresses of butyrate in regulation of endocrine and nervous system have been made in nearest 2 years. Researchers conducted an *ex vivo* experiment by using the isolated perfused rat colon. Through luminal and especially vascular infusion of acetate, propionate and butyrate, they observed that acetate and butyrate increased colonic glucagon-like peptide-1 (GLP-1) secretion with increased intracellular cAMP concentrations but independent in FFAR2/FFAR3 activation. The results suggested that all blood circulation, nerve and paracrine might play a part in the SCFAs-stimulated GLP-1 secretion [34]. Another research demonstrated that SCFAs decreased food take by activating vagal afferent via intraperitoneal injection of three SCFA molecules (acetate, propionate and butyrate) in fasted mice and switching off the vagal afferents of hepatic branch and capsaicin-sensitive sensory nerves [35].

However, butyrate doesn’t always perform its merits. It should be mentioned that the effect of butyrate on proliferation of intestinal epithelial stem cells depends on the concentration of butyrate. Low-dose butyrate promoted intestinal epithelial proliferation but butyrate at physiologic concentration suppressed proliferation [36]. Dialectical attitude is necessary to assess the impact of butyrate on body health.

#### Bile acids

Bile acids (BAs) are produced by liver and assist intestine to digest dietary lipid. Meanwhile, BAs play a vital role in regulating metabolic balance and intestinal homeostasis. Several lines of evidence implicate that BAs disorder is related to various diseases, like *C. difficile* infection, IBD, primary biliary cholangitis and non-alcoholic steatohepatitis [37]. Generally, many *Clostridium* species are involved in the production of primary and secondary BAs in ileum and colon.

##### The formation of BAs

In our intestine, primary BAs mainly include chenodeoxycholate cholate and their conjugates with taurine and glycine. Secondary BAs mainly consist of lithocholate and deoxycholate, although over 20 different secondary BAs have been detected in adult human feces [38]. Primary BAs are produced in liver via cholesterol catabolism, deposited as conjugates in gall bladder and released into small intestine after food intake. Conjugated BAs can be deconjugated by ileal bacteria like *Bacteroides*, *Bifidobacterium*, *Clostridium* and *Lactobacillus* [38] and then metabolized to secondary BAs by *Clostridium* and *Eubacterium* through dehydroxylation in the distal ileum and colon. Nowadays, the *Clostridium* species including *C. scindens*, *C. hiranonis*, *C. hylemonae*, *C. sordelli* and so forth, have been reported to secret 7α-Hydroxysteroid dehydrogenases (7α-HSDHs) [38, 39] (Fig. 1). These *Clostridium* species producing primary and secondary BAs play a vital part in improving resistance to *C. difficile* infection [39, 40].

##### Chenodeoxycholate and secondary BAs inhibit *C. difficile* infection

A study conducted in 2013 showed that CamSA, a bile salt analog could block *C. difficile* spore germination in vitro [41]. Oral administration of *C. scindens*, which can produce 7α-HSDHs, could enhance the resistance to *C. difficile* by increasing the content of secondary BAs [39]. Afterward, accumulating evidence has shown that most primary BAs promoted *C. difficile* spore germination while chenodeoxycholate and secondary BAs restrained the growth of *C. difficile* vegetative cells [40]. But why are the effects of BAs on *C. difficile* spore germination and growth discriminatory obviously? What are the potential mechanisms herein?

##### Potential mechanism of BAs on *C. difficile* infection resistance

The effect of BAs on *C. difficile* infection resistance may be mediated by their recognition of intestinal receptors. Farnesoid X Receptor (FXR) recognized BAs and then regulated the synthesis, transport and recycle of BAs to maintain their appropriate concentrations in intestine [42]. Another receptor, G protein coupled bile acid receptor 5 (TGR5) also recognizes BAs [43]. TLR5 exerts anti-inflammation effects through inhibiting the secretion of the proinflammatory cytokines TNF-α and IL12 and inducing NO production to resist monocyte adhesion [44, 45]. However, both FXR and TGR5 recognizes primary and secondary BAs while only chenodeoxycholate and secondary BAs restrained the growth of *C. difficile* vegetative cells. Hence, there may be some undiscovered specific receptors to chenodeoxycholate and secondary BAs.

#### Protein and other substances metabolisms of *Clostridium* species

In general, excess protein and AA fermentation in hindgut is detrimental for our health. Too much ammonia could directly and indirectly damage the intestinal epithelial cells. But there are still some benefits from bacterial protein fermentation, especially *Clostridium* species. Speaking frankly, protein or AA-fermenting *Clostridium* species are both angels and demons to our health.

AA-fermenting *Clostridium* species have been divided into five groups according to their AA metabolic patterns. Recent researches have played much attention on the bacterial metabolism of tryptophan (Trp) because its metabolites, like indoleacetic acid and indolepropionic acid (IPA) [46, 47], exerted surprising effects on body health. Some strains of *Clostridium sporogenes* and *Clostridium cadaveris* could convert Trp to IPA, which was verified to reduce the intestinal permeability [48, 49], promote intestinal barrier function via Pregnane X Receptor and Toll-like Receptor 4 pathways [50] and scavenge reactive oxygen species to prevent Alzheimer’s disease [48]. With ongoing researches, more biologic activities of metabolites from Clostridial protein fermentation are expected.

Except protein and AA, other bioactive substances are also the substrates utilized by *Clostridium* species. It was verified that *Clostridium bifermentans* was the predominate bacterium in human feces to produce 1,2-*sn*-Diacylglycerols (DAGs) through fermenting phosphatidylcholine. The metabolite DAGs were the activators of protein kinase C, which could regulate colonic mucosal proliferation [51]. What’s more, species of *Clostridium* are the main force to utilize phenolics, like flavanones, isoflavones, flavonols and flavan-3-ols [52, 53]. Most bioactive metabolites from phenolics metabolism are of great benefit to our health.

## Pathogenicity of *Clostridium* species

In spite of many benefits provided by *Clostridium* species, most anaerobic infections were induced by *Clostridium*, like *C. perfringens*, *C. difficile* and *C. botulinum*. Hence, the potential risks should be on guard against carefully. Herein, we will introduce several vital pathogenic *Clostridium* species and their harms to our health, in order to keep away from potential pathogens when we utilize *Clostridium* species as probiotics.

### *C. perfringens*

*C. perfringens* produce 4 typing toxins α, β, ε, ι and are divided into types A to E according to the ability to produce these 4 toxins. Except 4 typing toxins, *C. perfringens* also produce extra toxins, like *C. perfringens* enterotoxin and necrotic enteritis B-like toxin. The toxin genes are located in both chromosome and plasmids and *C. perfringens* can transfer toxin genes via conjugation in most cases [10]. These toxins possess a variety of biologic activities, like neurotoxicity, hemolytic and enterotoxigenic activity and the major modes of action are pore-forming, ADP-ribosylating, phospholipase C activity and Ras-Glycosylating. Generally, *C. perfringens* infection can induce necrotizing enteritis, gas gangrene enterotoxemia, gas gangrene and so on, along with high mortality rate [10].

### *C. difficile*

*C. difficile* infection often occurs after antibiotics therapy [54–56]. Antibiotics can eliminate part of commensal bacteria in gut and then the opportunistic *C. difficile* breeds crazily dues to imbalance between microbiota and intestinal immune system.

*C. difficile* damages our digestive system, especially colon, via its toxins. *C. difficile* produces 2 kinds of toxins: toxin A and B, both of which have enterotoxin. And toxin B also has cytotoxin. They can monoglucosylate and inactivate Rho subfamily proteins, then resulting in colitis with diarrhea via inducing polymorphonuclear neutrophils chemotaxis and fluid secretion [11, 57].

### Other pathogenic *Clostridium* species

*C. butyricum*, *C. tertium* and *C. paraputrificum* were proposed associated to necrotizing enterocolitis in preterm neonates [58, 59]. *C. butyricum* type E was also found to result in intestinal toxemia botulism via botulinum-like toxin secretion [60]. What’s more, *C. cadaveris* could trigger Bacteremia [61]. And *C. chauvoei* could cause blackleg of ruminant [62].

In consequence, we need pay special attention on all of toxins and other pathogenic factors from unfriendly *Clostridium* species when we develop novel probiotics from *Clostridium* species.

## Effects of diets and physiologic state on *Clostridium* species

As a rule, the efficacy of probiotics usage in disease prevention and animal production are affected by diet and physiologic state of human and animals. Combined usage of probiotics and prebiotics could multiply the probiotic effects than single usage. Meanwhile, the supplementation of *Clostridium* spp. may be not appropriate in every stage of life and may only prevent several diseases to some degree. Therefore, The following contents will focus on the effects of diets and physiologic state on *Clostridium* species, to give us more inspiration for targeted *Clostridium* application.

### Diets

*Clostrdium* spp. can be simply classified into two groups: carbohydrate-fermenting and protein-fermenting *Clostrdium* spp. according to the preference of carbohydrate and protein fermentation. Carbohydrate and protein in diet can powerfully shape the *Clostridium* patten in gut. Next, we will discuss the effect of dietary carbohydrate, protein and other bioactivators on *Clostridium* species in gut, in order to choose suitable prebiotics for concomitant use with *Clostridium* spp..

#### Dietary polysaccharides

Generally, *Clostridium* prefers dietary carbohydrate, especially non-starch polysaccharides. The alternation of dietary polysaccharides could affect the amount of *Clostridium* species in gut. For instance, the diets enriched in different fibers, such as inulin, oligofructose, arabinoxylan, guar gum and resistant starch, all of which induced the enrichment of *Clostridium* cluster IV and XIVa representatives along with changed mucosal energy metabolism [63]. Additionally, dietary inulin-type fructans and arabinoxylan-oligosaccharides could not only directly promote the growth and reproduction of *Clostridium* species but also indirectly facilitate the acetate production from bifidobacterial strains fermentation to provide more substrates for *Clostridium* species to produce butyrate [26]. However, unreasonable doses and impertinent fibers would produce counterproductive results. A study showed that species of *Clostridium* clusters IV and XIVa were decreased in pigs consuming 63% amylose, suggesting that appropriate doses of fibers should be taken into consideration [64]. Moreover, different kinds of fiber exert different impact on *Clostridium* colonized in different intestinal niches. 1.3% alfalfa added in diets improved the proportion of *Clostridium* clusters XIVa species in digesta of proximal colon while 1% pure cellulose increased the abundance of *Clostridium* clusters XIVa species in distal colonic mucosa [65]. The possible cause of this phenomenon may be the different physical and chemical properties of these two fibers. More interestingly, some adverse effects emerged in several experiments. 5% soybean hulls added in ration of weaned piglets reduced the proportion of *Anaerofilum*, *norank_-f_Ruminococcaceae*, and *Eubacterium_ventriosum_group* in feces [66]. It is reasonable to certain degree because of high-content anti-nutritional factors in soybean hulls and turbulent intestinal environment of piglet under weaning stress, though soybean hulls has higher total non-starch polysaccharides than same-weight corn bran and wheat bran.

As a whole, the benefits of dietary polysaccharides on abundance of *Clostridium* species depend on the type, dose of fibers and growth stage of animals or human.

#### Other nutrients and bioactivators

Fecal *Clostridium* Cluster IV and XIVa species were reported decreasing in highly digestible casein and the less digestible, fiber-rich soybean meal [67, 68]. Besides, low-level protein diet applied in finishing pigs (from 16% to 13% crude protein) induced decrement of the proportion of *Clostridium _sensu_stricto_1* in ileum (from 44.76% to 19.92%) while the abundance of *Clostridium _sensu_stricto_1* in colon increased (from 6.86% to 19.00%) along with the protein concentration reduction [69]. Herein, *Clostridium _sensu_stricto_1* refers to the *Clostridium* cluster I. The change of their proportion in colon is strange and possibly dues to the high proportion of unidentified bacteria on genus level (42.67% and 50.66% in 2 groups respectively).

Part of polyphenols can be degraded by some *Clostridium* species and the polyphenol content in diet also affect the abundance of *Clostridium* species in turn. Supplementations of polyphenol-rich grape pomace concentrate (60 g/kg) and grape seed extract (7.2 g/kg) in the diet of broiler chicks decreased the proportion of *Clostridium* species in ileal digesta while higher proportion of *Clostridium* species were found in cecal digesta [70]. The inmost mechanism behind the opposite results in different intestinal segment is needed to explore with more efforts. In addition, trehalose, as a kind of food additive in our life, enhanced the virulence of a *C. difficile* epidemic ribotype strain [71], suggesting that our lifestyle plays an outstanding role in alteration of *Clostridium* species pattern in our gut.

### Physiologic state of body

Except diets, the physiologic state of body conspicuously affects the abundance of *Clostridium* species in gut. Diseases can induce a collapse of the intestinal microbial community structure, including *Clostridium* species.

When mice were infected by *Salmonella typhimurium*, the dysbiosis of intestinal microbiota would emerge quickly [72]. Then the bacteria of Clostridia were decreased with decrement of butyrate and increment of lactate, which was utilized by *S. typhimurium* to enhance its invasion [73]. Fructose-Asparagine is another vital nutrient for *S. typhimurium* to exert pro-inflammation effects and *S. typhimurium* competed for it with *Clostridium* species. The successful invasion and proliferation of *S. typhimurium* in gut meant that *Clostridium* species were defeated with lower abundance in gut [74]. The count of *Clostridium* clusters III, IV, and XIVa also reduced in intestinal failure [8]. Further, *Roseburia hominis* and *F. prausnitzii* were decreased in ulcerative colitis patients [71]. But in allergic sensitization, eczema, or asthma, there was higher abundance of Clostridiaceae along with increased Bacteroidaceae and Enterobacteriaceae [75]. Therefore, we should take the physiologic state of body into consideration when we prevent or treat different diseases with *Clostridium* species.

## Potentiality and challenges of *Clostridium* species as probiotics

### Potentiality

On the basis of the above analyses, the advantages of *Clostridium* as potential probiotics are concluded below. Firstly, *Clostridium* species are the commensal bacteria in the gut of animals and human. They are affable to human and animals and can’t trigger strong intestinal immune response. Secondly, most *Clostridium* species can sporulate and successfully resist on stressful environments. Thirdly, *Clostridium* species, especially *Clostridium* cluster XIVa and IV species, can exert anti-inflammation effects and maintain the intestinal health via their components and metabolites, especially butyrate. Hence, *Clostridium* species as probiotics has a broad prospect in the future.

However, despite that, there are still some challenges in application of *Clostridium* species to improve health of human and animals.

### Challenges

#### Safety

Recombination and insertion of botulinum neurotoxin complex genes were discovered in some *C. botulinum* and *C. butyricum* type E strains [76]. Toxins plasmids of *C. perfringens* were discovered in other commensal bacteria in gut [10]. So *Clostridium* species must be detected strictly through safety assessment of probiotic strains. Toxin genes should be excluded to avoid vertical and horizontal transmission of virulence factors. Except that, antibiotics resistance genes should be taken into consideration conventionally. Furthermore, carbohydrate-fermentating *Clostridium* is preferred to avoid possible harmful effects of protein or AA fermentation.

#### Efficiency

Nowadays, the whole area of probiotics development is full of uncertainty [77]. Most probiotic trials have limitations because of their poor methodologic quality [78]. And the effects of some probiotics are uneven and vary in individuals. A scientist even proposed that development of one-size-fits-all probiotic was unpractical [79]. As for *Clostridium* species, there are at least five challenges in improving their efficiencies in medical interference and animal husbandry.

Firstly, powerful adhesion in intestinal surface is vital to hold everlasting and consistent benefits, so it is wise to select the *Clostridium* strains which possess high adhesion ability as candidates. Secondly, give priority to spore-forming *Clostridium* species, which have strong stress-resistant ability. We should try our best to improve the total spore count *in vitro* and germination rate *in vivo*. But until now, most studies involved in how *Clostridium* spp. sporulate and germinate prefered to *C. difficile* and *C. perfringens* and many key questions still remain unanswered. Meanwhile, *C. difficile* only have 25% homologs of spore coat proteins in *Bacillus subtilis*, whose spore biology is well-studied [80]. Therefore it is urgent to uncover the underlying mechanisms of *Clostridium* spp. sporulation and germination with more in-depth studies. Thirdly, advocate combined utilization of different *Clostridium* strains or *Clostridium* spp. and other probiotics or prebiotics, such as combination of *Clostridium* spp. and *Bifidobacterium* spp. (cross-feeding) or combination of *Clostridium* spp. and dietary fiber (the preferred nutrients for *Clostridium* spp.) [81, 82]. This strategy tallys with the idea of microbial ecosystems therapeutics, which utilizes a mixture of defined bacteria or core microbiome to treat diseases [83]. Several researches with this strategy obtained positive results in some experiments and clinic trials, although more large-scale trials are required to confirm its efficiency [84, 85]. Forthly, seriously consider the individual differences, like dietary habit, age, physiologic state, previous microbial community and growth stage of animals, in order to improve applicability of *Clostridium* species. A study showed that the increment of *Clostridum* spp. in gut could maintain the “lean” phenotype of human or animals via inhibiting the expression of lipid absorption-related genes [86]. So it may be wiser to apply *Clostridium* spp. in improving the gut health of young or breeding animals, rather than animal production performance. Lastly, take specie and strain-specificity into consideration. The probiotic effects vary among different species and strains of *Clostridium*. Hence, we should evaluate them case by case.

All in all, the future of *Clostridium* species developed as probiotics is hopeful but tortuous.

## Conclusion

*Clostridium* species, as the outstanding representative of intestinal commensal bacteria, possess potent probiotic characteristics for intestinal homeostasis. In spite of some risks like toxins release and some challenges in application, *Clostridium* species still have a rosiness future as a member of probiotic family. And more valid researches will accelerate the development and achievement of *Clostridium* species as probiotics in the future.

## Data Availability

The data were shown in the main manuscript and available to readers.

## Abbreviations

7α-HSDHs: 7α-Hydroxysteroid dehydrogenases
AAs: Amino acids
*C. butyricum*: *Clostridium butyricum*
*C. difficile*: *Clostridium difficile*
*C. perfringens*: *Clostridium perfringens*
DAG: 1,2-*sn*-Diacylglycerols
EPM: Extracellular polymeric matrix
FXR: Farnesoid X Receptor
GLP-1: Glucagon-like peptide-1
IBD: Inflammatory bowel disease
IPA: Indolepropionic acid
SCFAs: Short-chain fatty acids
TGR5: G protein coupled bile acid receptor 5
Trp: Tryptophan

## References

[1] Amitay EL, Krilaviciute A, Brenner H (2018). Systematic review: gut microbiota in fecal samples and detection of colorectal neoplasms. Gut Microbes.

[2] Arnoldini M, Cremer J, Hwa T (2018). Bacterial growth, flow, and mixing shape human gut microbiota density and composition. Gut Microbes.

[3] Flemer B, Herlihy M, O'Riordain M, Shanahan F, O'Toole PW (2018). Tumour-associated and non-tumour-associated microbiota: addendum. Gut Microbes.

[4] Gomes AC, Hoffmann C, Mota JF (2018). The human gut microbiota: metabolism and perspective in obesity. Gut Microbes.

[5] Nagano Y, Itoh K, Honda K (2012). The induction of Treg cells by gut-indigenous *Clostridium*. Curr Opin Immunol.

[6] Momose Y, Maruyama A, Iwasaki T, Miyamoto Y, Itoh K (2009). 16S rRNA gene sequence-based analysis of clostridia related to conversion of germfree mice to the normal state. J Appl Microbiol.

[7] Shah R, Cope JL, Nagy-Szakal D, Dowd S, Versalovic J, Hollister EB (2016). Composition and function of the pediatric colonic mucosal microbiome in untreated patients with ulcerative colitis. Gut Microbes.

[8] Till H, Castellani C, Moissl-Eichinger C, Gorkiewicz G, Singer G (2015). Disruptions of the intestinal microbiome in necrotizing enterocolitis, short bowel syndrome, and Hirschsprung's associated enterocolitis. Front Microbiol.

[9] Atarashi K, Tanoue T, Oshima K, Suda W, Nagano Y, Nishikawa H (2013). Treg induction by a rationally selected mixture of clostridia strains from the human microbiota. Nature.

[10] Li J, Adams V, Bannam TL, Miyamoto K, Garcia JP, Uzal FA (2013). Toxin plasmids of *Clostridium perfringens*. Microbiol Mol Biol Rev.

[11] Songer JG (2010). Clostridia as agents of zoonotic disease. Vet Microbiol.

[12] Gomes AC, Hoffmann C, Mota JF (2018). The human gut microbiota: metabolism and perspective in obesity. Gut Microbes.

[13] Albenberg LG, Wu GD (2014). Diet and the intestinal microbiome: associations, functions, and implications for health and disease. Gastroenterology.

[14] Alipour MJ, Jalanka J, Pessa-Morikawa T, Kokkonen T, Satokari R, Hynonen U (2018). The composition of the perinatal intestinal microbiota in cattle. Sci Rep.

[15] Sato R, Tanaka M (1997). Intestinal distribution and intraluminal localization of orally administered *Clostridium butyricum* in rats. Microbiol Immunol.

[16] Hachisuka Y, Suzuki I, Morikawa K, Maeda S (1982). The effect of oxidation-reduction potential on spore germination, outgrowth, and vegetative growth of *Clostridium tetani*, *Clostridium butyricum*, and *Bacillus subtilis*. Microbiol Immunol.

[17] Sinji K, Tomoyuki N, Yoshitaka N, Yoshimi B, Tai U, Kazuo K (1998). Effect of oxygen on the growth of *Clostridium butyricum* (type species of the genus *Clostridium*), and the distribution of enzymes for oxygen and for active oxygen species in clostridia. J Ferment Bioeng.

[18] Khan MT, Duncan SH, Stams AJM, van Dijl JM, Flint HJ, Harmsen HJM (2012). The gut anaerobe *Faecalibacterium prausnitzii* uses an extracellular electron shuttle to grow at oxic–anoxic interphases. ISME J.

[19] Umesaki Y, Setoyama H, Matsumoto S, Imaoka A, Itoh K (1999). Differential roles of segmented filamentous bacteria and clostridia in development of the intestinal immune system. Infect Immun.

[20] Pan X, Wu T, Zhang L, Song Z, Tang H, Zhao Z (2008). In vitro evaluation on adherence and antimicrobial properties of a candidate probiotic *Clostridium butyricum* CB2 for farmed fish. J Appl Microbiol.

[21] Sokol H, Pigneur B, Watterlot L, Lakhdari O, Bermúdez-Humarán LG, Gratadoux J-J (2008). Faecalibacterium prausnitzii is an anti-inflammatory commensal bacterium identified by gut microbiota analysis of Crohn disease patients. Proc Natl Acad Sci.

[22] Martin R, Chain F, Miquel S, Lu J, Gratadoux J-J, Sokol H (2014). The commensal bacterium *Faecalibacterium prausnitzii* is protective in DNBS-induced chronic moderate and severe colitis models. Inflamm Bowel Dis.

[23] Kiely CJ, Pavli P, O'Brien CL (2018). The role of inflammation in temporal shifts in the inflammatory bowel disease mucosal microbiome. Gut Microbes.

[24] Richard ML, Liguori G, Lamas B, Brandi G, da Costa G, Hoffmann TW (2018). Mucosa-associated microbiota dysbiosis in colitis associated cancer. Gut Microbes.

[25] Atarashi K, Tanoue T, Shima T, Imaoka A, Kuwahara T, Momose Y (2011). Induction of colonic regulatory T cells by indigenous *Clostridium* species. Science.

[26] Godefroy E, Alameddine J, Montassier E, Mathé J, Desfrançois-Noël J, Marec N (2018). Expression of CCR6 and CXCR6 by gut-derived CD4/CD8α T-regulatory cells, which are decreased in blood samples from patients with inflammatory bowel diseases. Gastroenterology.

[27] Tong X, Xu J, Lian F, Yu X, Zhao Y, Xu L (2018). Structural alteration of gut microbiota during the amelioration of human type 2 diabetes with hyperlipidemia by metformin and a traditional Chinese herbal formula: a multicenter, randomized, open label clinical trial. MBio.

[28] Miquel S, Leclerc M, Martin R, Chain F, Lenoir M, Raguideau S, et al. Identification of metabolic signatures linked to anti-inflammatory effects of Faecalibacterium prausnitzii. MBio. 2015;6:e00300–15. 10.1128/mBio.00300-15 PMC4453580 25900655

[29] Riviere A, Selak M, Lantin D, Leroy F, De Vuyst L (2016). Bifidobacteria and butyrate-producing colon bacteria: importance and strategies for their stimulation in the human gut. Front Microbiol.

[30] Luo H, Yang R, Zhao Y, Wang Z, Liu Z, Huang M (2018). Recent advances and strategies in process and strain engineering for the production of butyric acid by microbial fermentation. Bioresour Technol.

[31] O'Keefe SJD (2016). Diet, microorganisms and their metabolites, and colon cancer. Nat Rev Gastroenterol Hepatol.

[32] Liu H, Wang J, He T, Becker S, Zhang G, Li D (2018). Butyrate: a double-edged sword for health?. Adv Nutr.

[33] Huang C, Song P, Fan P, Hou C, Thacker P, Ma X (2015). Dietary sodium butyrate decreases Postweaning diarrhea by modulating intestinal permeability and changing the bacterial communities in weaned piglets. J Nutr.

[34] Christiansen CB, Gabe MBN, Svendsen B, Dragsted LO, Rosenkilde MM, Holst JJ (2018). The impact of short-chain fatty acids on GLP-1 and PYY secretion from the isolated perfused rat colon. Am J Physiol Gastrointest Liver Physiol.

[35] Goswami C, Iwasaki Y, Yada T (2018). Short-chain fatty acids suppress food intake by activating vagal afferent. J Nutr Biochem.

[36] Kaiko GE, Ryu SH, Koues OI, Collins PL, Solnica-Krezel L, Pearce EJ (2016). The colonic crypt protects stem cells from microbiota-derived metabolites. Cell.

[37] Hegyi P, Maléth J, Walters JR, Hofmann AF, Keely SJ (2018). Guts and gall: bile acids in regulation of intestinal epithelial function in health and disease. Physiol Rev.

[38] Gérard P (2014). Metabolism of cholesterol and bile acids by the gut microbiota. Pathogens..

[39] Buffie CG, Bucci V, Stein RR, McKenney PT, Ling L, Gobourne A (2014). Precision microbiome reconstitution restores bile acid mediated resistance to *Clostridium difficile*. Nature.

[40] Shen A (2015). A gut odyssey: the impact of the microbiota on *Clostridium difficile* spore formation and germination. PLoS Pathog.

[41] Howerton A, Patra M, Abel-Santos E (2013). A new strategy for the prevention of *Clostridium difficile* infection. J Infect Dis.

[42] Bashan A, Gibson TE, Friedman J, Carey VJ, Weiss ST, Hohmann EL (2016). Universality of human microbial dynamics. Nature.

[43] Makki K, Deehan EC, Walter J, Bäckhed F (2018). The impact of dietary fiber on gut microbiota in host health and disease. Cell Host Microbe.

[44] Högenauer K, Arista L, Schmiedeberg N, Werner G, Jaksche H, Bouhelal R (2014). G-protein-coupled bile acid receptor 1 (GPBAR1, TGR5) agonists reduce the production of proinflammatory cytokines and stabilize the alternative macrophage phenotype. J Med Chem.

[45] Catry Emilie, Bindels Laure B, Tailleux Anne, Lestavel Sophie, Neyrinck Audrey M, Goossens Jean-François, Lobysheva Irina, Plovier Hubert, Essaghir Ahmed, Demoulin Jean-Baptiste, Bouzin Caroline, Pachikian Barbara D, Cani Patrice D, Staels Bart, Dessy Chantal, Delzenne Nathalie M (2017). Targeting the gut microbiota with inulin-type fructans: preclinical demonstration of a novel approach in the management of endothelial dysfunction. Gut.

[46] Whitehead TR, Price NP, Drake HL, Cotta MA (2008). Catabolic pathway for the production of skatole and indoleacetic acid by the acetogen *Clostridium drakei*, *Clostridium scatologenes*, and swine manure. Appl Environ Microbiol.

[47] Dickert S, Pierik AJ, Buckel W (2002). Molecular characterization of phenyllactate dehydratase and its initiator from *Clostridium sporogenes*. Mol Microbiol.

[48] Li H. (R)-Indolelactyl-CoA dehydratase, the key enzyme of tryptophan reduction to indolepropionate in *Clostridium sporogenes*: Philipps-Universität Marburg; 2014.

[49] Ma N, Guo P, Zhang J, He T, Kim SW, Zhang G (2018). Nutrients mediate intestinal bacteria-mucosal immune crosstalk. Front Immunol.

[50] Venkatesh M, Mukherjee S, Wang H, Li H, Sun K, Benechet AP (2014). Symbiotic bacterial metabolites regulate gastrointestinal barrier function via the xenobiotic sensor PXR and toll-like receptor 4. Immunity.

[51] Vulevic J, McCartney AL, Gee JM, Johnson IT, GIBSON GR (2004). Microbial species involved in production of 1,2-sn-diacylglycerol and effects of phosphatidylcholine on human fecal microbiota. Appl Environ Microbiol.

[52] Yokoyama S-I, Oshima K, Nomura I, Hattori M, Suzuki T (2011). Complete genomic sequence of the O-Desmethylangolensin-producing bacterium *Clostridium* rRNA cluster XIVa strain SY8519, isolated from adult human intestine. J Bacteriol.

[53] Selma MV, Espin JC, Tomas-Barberan FA (2009). Interaction between phenolics and gut microbiota: role in human health. J Agric Food Chem.

[54] Markey L, Shaban L, Green ER, Lemon KP, Mecsas J, Kumamoto CA (2018). Pre-colonization with the commensal fungus *Candida albicans* reduces murine susceptibility to *Clostridium difficile* infection. Gut Microbes.

[55] Sokol H, Jegou S, McQuitty C, Straub M, Leducq V, Landman C (2018). Specificities of the intestinal microbiota in patients with inflammatory bowel disease and *Clostridium difficile* infection. Gut Microbes.

[56] Zackular JP, Skaar EP (2018). The role of zinc and nutritional immunity in *Clostridium difficile* infection. Gut Microbes.

[57] Anjuwon-Foster BR, Tamayo R (2018). Phase variation of *Clostridium difficile* virulence factors. Gut Microbes.

[58] Azcarate-Peril MA, Foster DM, Cadenas MB, Stone MR, Jacobi SK, Stauffer SH (2011). Acute necrotizing enterocolitis of preterm piglets is characterized by dysbiosis of ileal mucosa-associated bacteria. Gut Microbes.

[59] Kiu R, Caim S, Alcon-Giner C, Belteki G, Clarke P, Pickard D (2017). Preterm infant-associated *Clostridium tertium*, *Clostridium cadaveris*, and *Clostridium paraputrificum* strains: genomic and evolutionary insights. Genome Biol Evol.

[60] Fenicia L, Franciosa G, Pourshaban M, Aureli P (1999). Intestinal toxemia botulism in two young people, caused by *Clostridium butyricum* type E. Clin Infect Dis.

[61] Schade RP, Van Rijn M, Timmers HJLM, Dofferhoff ASM, Klaassen CHW, Meis JFGM (2006). Clostridium cadaveris bacteraemia: two cases and review. Scand J Infect Dis.

[62] Pires PS, Santos RL, da Paixao TA, de Oliveira Bernardes LC, de Macedo AA, Goncalves LA (2017). Intracellular survival of *Clostridium chauvoei* in bovine macrophages. Vet Microbiol.

[63] Lange K, Hugenholtz F, Jonathan MC, Schols HA, Kleerebezem M, Smidt H (2015). Comparison of the effects of five dietary fibers on mucosal transcriptional profiles, and luminal microbiota composition and SCFA concentrations in murine colon. Mol Nutr Food Res.

[64] Fouhse JM, Ganzle MG, Regmi PR, van Kempen TATG, Zijlstra RT (2015). High amylose starch with low *in vitro* digestibility stimulates hindgut fermentation and has a bifidogenic effect in weaned pigs. J Nutr.

[65] Mu C, Zhang L, He X, Smidt H, Zhu W (2017). Dietary fibres modulate the composition and activity of butyrate-producing bacteria in the large intestine of suckling piglets. Antonie Van Leeuwenhoek.

[66] Zhao J, Liu P, Wu Y, Guo P, Liu L, Ma N (2018). Dietary fiber increases butyrate-producing bacteria and improves the growth performance of weaned piglets. J Agric Food Chem.

[67] Rist VTS, Weiss E, Sauer N, Mosenthin R, Eklund M (2014). Effect of dietary protein supply originating from soybean meal or casein on the intestinal microbiota of piglets. Anaerobe..

[68] Pieper R, Kröger S, Richter JF, Wang J, Martin L, Bindelle J (2012). Fermentable fiber ameliorates fermentable protein-induced changes in microbial ecology, but not the mucosal response, in the colon of piglets. J Nutr.

[69] Fan P, Liu P, Song P, Chen X, Ma X (2017). Moderate dietary protein restriction alters the composition of gut microbiota and improves ileal barrier function in adult pig model. Sci Rep.

[70] Biasi F, Deiana M, Guina T, Gamba P, Leonarduzzi G, Poli G (2014). Wine consumption and intestinal redox homeostasis. Redox Biol.

[71] Collins J, Robinson C, Danhof H, Knetsch CW, van Leeuwen HC, Lawley TD (2018). Dietary trehalose enhances virulence of epidemic *Clostridium difficile*. Nature.

[72] Zhang Y-G, Singhal M, Lin Z, Manzella C, Kumar A, Alrefai WA (2018). Infection with enteric pathogens *Salmonella typhimurium* and *Citrobacter rodentium* modulate TGF-beta/Smad signaling pathways in the intestine. Gut Microbes.

[73] Gillis CC, Hughes ER, Spiga L, Winter MG, Zhu W, Furtado de Carvalho T (2018). Dysbiosis-associated change in host metabolism generates lactate to support *Salmonella* growth. Cell Host Microbe.

[74] Wu J, Sabag-Daigle A, Borton MA, Kop LFM, Szkoda BE, Kaiser BLD, et al. *Salmonella*-mediated inflammation eliminates competitors for fructose-asparagine in the gut. Infect Immun. 2018;86. 10.1128/IAI.00945-17 PMC5913863 29483291

[75] Zimmermann P, Messina N, Mohn WW, Finlay BB, Curtis N (2019). Association between the intestinal microbiota and allergic sensitization, eczema, and asthma: a systematic review. J Allergy Clin Immunol.

[76] Hill KK, Xie G, Foley BT, Smith TJ, Munk AC, Bruce D (2009). Recombination and insertion events involving the botulinum neurotoxin complex genes in *Clostridium botulinum* types A, B, E and F and *Clostridium butyricum* type E strains. BMC Biol.

[77] Sanders ME, Younes J (2018). Paper incompletely describes evidence-based usage of probiotics. Gut Microbes.

[78] Koretz RL (2018). Probiotics in gastroenterology: how pro is the evidence in adults?. Am J Gastroenterol.

[79] Yi SH, Jernigan JA, McDonald LC (2016). Prevalence of probiotic use among inpatients: a descriptive study of 145 U.S. hospitals. Am J Infect Control.

[80] Daniel P-S, Aimee S, Joseph AS (2014). *Clostridium difficile* spore biology: sporulation, germination, and spore structural proteins. Trends Microbiol.

[81] Ibarra A, Latreille-Barbier M, Donazzolo Y, Pelletier X, Ouwehand AC (2018). Effects of 28-day *Bifidobacterium animalis* subsp. lactis HN019 supplementation on colonic transit time and gastrointestinal symptoms in adults with functional constipation: a double-blind, randomized, placebo-controlled, and dose-ranging trial. Gut Microbes.

[82] Solano-Aguilar G, Shea-Donohue T, Madden KB, Quinoñes A, Beshah E, Lakshman S (2018). *Bifidobacterium animalis* subspecies lactis modulates the local immune response and glucose uptake in the small intestine of juvenile pigs infected with the parasitic nematode *Ascaris suum*. Gut Microbes.

[83] Petrof EO, Claud EC, Gloor GB, Allen-Vercoe E (2013). Microbial ecosystems therapeutics: a new paradigm in medicine?. Benefic Microbes.

[84] Petrof EO, Gloor GB, Vanner SJ, Weese SJ, Carter D, Daigneault MC (2013). Stool substitute transplant therapy for the eradication of *Clostridium difficile* infection: “RePOOPulating” the gut. Microbiome.

[85] Martz S-LE, McDonald JAK, Sun J, Zhang Y-G, Gloor GB, Noordhof C (2015). Administration of defined microbiota is protective in a murine *Salmonella* infection model. Sci Rep.

[86] Wang Y, Hooper LV (2019). Immune control of the microbiota prevents obesity. Science..

